# Vascular autorescaling of fMRI (VasA fMRI) improves sensitivity of population studies: A pilot study

**DOI:** 10.1016/j.neuroimage.2015.09.033

**Published:** 2016-01-01

**Authors:** Samira M. Kazan, Siawoosh Mohammadi, Martina F. Callaghan, Guillaume Flandin, Laurentius Huber, Robert Leech, Aneurin Kennerley, Christian Windischberger, Nikolaus Weiskopf

**Affiliations:** a Wellcome Trust Centre for Neuroimaging, UCL Institute of Neurology, University College London, London WC1N 3BG, United Kingdom; b NMR-Unit, Max Planck Institute for Human Cognition and Brain Sciences, Leipzig, Germany; c Cognitive, Clinical and Computational Neuroimaging Lab, Imperial College, Hammersmith Hospital, University of London, London W12 0NN, United Kingdom; d Department of Psychology, University of Sheffield, Western Bank, Sheffield S10 2TN, United Kingdom; e MR Centre of Excellence, Centre for Medical Physics and Biomedical Engineering, Medical University of Vienna, Waehringer Guertel 18-20, Vienna A-1090, Austria; f Department of Neurophysics, Max Planck Institute for Human Cognitive and Brain Sciences, Leipzig, Germany

**Keywords:** BOLD fMRI, Group analysis, Vascularization differences, Autorescaling, ALFF

## Abstract

The blood oxygenation level-dependent (BOLD) signal is widely used for functional magnetic resonance imaging (fMRI) of brain function in health and disease. The statistical power of fMRI group studies is significantly hampered by high inter-subject variance due to differences in baseline vascular physiology. Several methods have been proposed to account for physiological vascularization differences between subjects and hence improve the sensitivity in group studies. However, these methods require the acquisition of additional reference scans (such as a full resting-state fMRI session or ASL-based calibrated BOLD). We present a vascular autorescaling (VasA) method, which does not require any additional reference scans. VasA is based on the observation that slow oscillations (< 0.1 Hz) in arterial blood CO_2_ levels occur naturally due to changes in respiration patterns. These oscillations yield fMRI signal changes whose amplitudes reflect the blood oxygenation levels and underlying local vascularization and vascular responsivity. VasA estimates proxies of the amplitude of these CO_2_-driven oscillations directly from the residuals of task-related fMRI data without the need for reference scans. The estimates are used to scale the amplitude of task-related fMRI responses, to account for vascular differences. The VasA maps compared well to cerebrovascular reactivity (CVR) maps and cerebral blood volume maps based on vascular space occupancy (VASO) measurements in four volunteers, speaking to the physiological vascular basis of VasA. VasA was validated in a wide variety of tasks in 138 volunteers. VasA increased *t*-scores by up to 30% in specific brain areas such as the visual cortex. The number of activated voxels was increased by up to 200% in brain areas such as the orbital frontal cortex while still controlling the nominal false-positive rate. VasA fMRI outperformed previously proposed rescaling approaches based on resting-state fMRI data and can be readily applied to any task-related fMRI data set, even retrospectively.

## Highlights

• We present a vascular autocalibration method (VasA) for fMRI studies.
• VasA fMRI accounts for physiological vascularization differences between subjects.
• VasA fMRI significantly increases functional sensitivity in group analyses.
• VasA does not require additional reference scans or other complicated procedures.
• VasA can be readily applied to any task-related fMRI data set, even retrospectively.

## Introduction

Functional magnetic resonance imaging (fMRI) is a non-invasive brain imaging technique that offers high spatial and temporal resolution. As such, it enables studies of brain function segregation and integration in large groups and allows for inferences about cognitive function at the population level.

The great majority of fMRI studies are based on the blood oxygenation level-dependent (BOLD) effect. The measured BOLD response indirectly reflects the underlying neuronal activity (Logothetis, 2008). It depends on a complex interaction between changes in cerebral blood flow (CBF), blood volume (CBV), and blood oxygenation (Buxton et al., 2004) that are coupled to neuronal activity (Villringer and Dirnagl, 1995, Logothetis and Wandell, 2004, Lauritzen, 2005). The baseline of these different physiological parameters varies across different individuals and across different brain regions. Such variations affect the BOLD response amplitude and cerebrovascular reactivity (CVR) (Ainslie and Duffin, 2009). Thus, the variations increase the inter-individual variability beyond differences in neuronal processing and consequently reduce the statistical power of fMRI group studies (D'Esposito et al., 1999, Huettel and McCarthy, 2001).

The low sensitivity of fMRI group studies and concomitant high false-negative rate (Type II error) is recognized as a central issue (Lieberman and Cunningham, 2009) since it obscures small effects as frequently encountered in cognitive and emotion processing. Attempts to increase the sensitivity have included improving the signal-to-noise ratio (SNR) of fMRI by, e.g., improved radio-frequency (RF) receive coils (Wiggins et al., 2006) or increased static magnetic fields (Yang et al., 1999, Fera et al., 2004). Post-processing techniques have also been used to reduce the impact of physiological noise (Hutton et al., 2011), which becomes more important with higher image SNR (Triantafyllou et al., 2005, Hutton et al., 2011). All approaches effectively increased the BOLD sensitivity in the individual, but the increased functional sensitivity at the single subject level only partly translated into an increased statistical power in group analyses. For example, increasing the field strength from 1.5 T to 3 T improved the functional sensitivity by only up to 30% in group analyses as quantified by t-value increases (Kruger et al., 2001, Krasnow et al., 2003, Garcia-Eulate et al., 2011), which is much less than the improvements of ~ 100% observed in single subject analyses (e.g., 1.5 T vs. 4 T in (Gati et al., 1997)). Although increasing the number of RF receive coils and channels from 12 to 32 increased the sensitivity in single subject analyses by about 25% (Kaza et al., 2011), again only small and equivocal differences were found in group analyses (Kaza et al., 2011).

The disappointingly small increases in functional sensitivity in group studies, despite the significant increases in sensitivity at the individual subject level, is related to inter-individual biological variance rather than noise in the data acquisition. Differences in structural anatomy and functional organization across individuals are also important sources of variation (Mueller et al., 2013), even when state of the art inter-subject registration methods or spatial normalization methods are used (Ashburner and Friston, 2011). This study addresses the inter-individual differences in vascularization, which is one major source of inter-individual biological variance. In particular, the amplitude of the BOLD response varies significantly across the population, i.e., the same level of neuronal activity can generate different BOLD signal amplitudes in different individuals. Previous approaches mapped and calibrated for these vascular response differences using separate reference scans based on CBF measurements, hypercapnia experiments, or resting-state fMRI experiments (rsfMRI) (Bandettini and Wong, 1997, Kannurpatti and Biswal, 2008, Kalcher et al., 2013).

All calibration methods address the complication that the BOLD fMRI signal is a measure of the hemodynamic activity in the brain and serves as an indirect indicator of the neural processes, which are the primary focus of most functional studies. It is therefore necessary to account for the hemodynamic responsivity to reveal the underlying neural interactions and make inferences. One established method to untangle these interactions is calibrated BOLD (Davis et al., 1998). The Davis model represents the foundation of these calibration methods where the BOLD signal is represented by a non-linear interaction between the fractional changes in CBF and cerebral metabolic rate of oxygen (CMRO_2_) multiplied by a vascularization parameter *M* (Davis et al., 1998). The parameter *M* (see Materials and Methods, Theory, Materials and Methods, Theory), is multiplicative and hence can be used to calibrate a voxel's BOLD signal. Because neuronal-driven BOLD signal changes are influenced by the same vascular mechanisms as CO_2_-driven changes (Bandettini and Wong, 1997, Davis et al., 1998, Cohen et al., 2004), the *M* parameter can be estimated by manipulating CO_2_ levels. However, this requires additional scans for manipulating CO_2_ levels, often involving respiratory challenges (e.g., Hypercapnia—increased fractional inspired carbon dioxide, FiCO_2_), which are impractical in many situations. They can be particularly stressful for patients or impossible in some disease conditions.

We propose a vascular autorescaling (VasA) method that maximizes the functional sensitivity in population studies without the need for any additional reference scans. VasA fMRI is based on the concept that changes in breathing patterns induce slow (< 0.1 Hz) variations in arterial blood CO_2_ levels (Van den Aardweg and Karemaker, 2002, Wise et al., 2004, Chang and Glover, 2009). The variations in blood CO_2_ levels cause significant changes in the amplitude of the BOLD signal. Wise et al. (2004) showed that the resting-state fluctuation amplitude (RSFA) %BOLD oscillations of around 0.2%, while for 5% CO_2_ %BOLD signal changes are around 2–3% (Yezhuvath et al., 2009, Donahue et al., 2014) and similarly for breath-hold paradigms (Murphy et al., 2011) at 3 T. The innovation of VasA lies in the realization that this vascularization map may be derived from the residuals of the task-related fMRI (tfMRI) data after removing task-related variance and slow drifts.

Previously, the power estimates of the CO_2_-driven low-frequency fluctuations (0.01–0.08 Hz) were extracted from rsfMRI reference scans as a vascular marker by (Di et al., 2013, Kalcher et al., 2013)—named Amplitude of low-frequency fluctuations (ALFF) or (conceptually identical) RSFA. These estimates were then used to scale the amplitude of tfMRI data in the same individual (Kannurpatti and Biswal, 2008, Kannurpatti et al., 2012, Tsvetanov et al., 2015). The proposed VasA method extracts similar ALFF maps directly from the tfMRI data and thus makes additional reference scans unnecessary and, importantly, can be applied to any data set, even retrospectively. We note that the calculation of ALFF was originally introduced by Zang et al. (2007) studying baseline activity in ADHD but is used here and in the previous studies as a vascular marker (Di et al., 2013, Kalcher et al., 2013, Tsvetanov et al., 2015).

We apply the VasA fMRI analysis technique to a variety of large fMRI data sets including different tasks and acquisition protocols (Price et al., 2010, Barch et al., 2013, Glasser et al., 2013, Larson-Prior et al., 2013). We demonstrate that it increases sensitivity at the group level as reflected in an increase of activated voxels by up to 42% for specific tasks and an increase of local *t*-scores of more than 30%, while retaining full control of false positives (Type I error). Comparisons of VasA maps to CVR and CBV maps suggest that VasA captured the intra- and inter-individual vascularization differences in BOLD fMRI.

## Materials and methods

### Theory

The BOLD fMRI signal depends on the complex coupling between neuronal activity, changes in cerebral blood flow, blood volume, and oxygenation in the activated brain regions (Buxton et al., 2004). Changes in BOLD signal amplitude can be directly related to variations in CBF, CBV, and CMRO_2_ or combinations of these (Davis et al., 1998). The relationship between these parameters can be expressed analytically using the Davis model (Davis et al., 1998). If we define the BOLD fMRI signal change with activation (*ΔS*_act_) as the difference between the resting state (*S*_rest_) and activated state (*S*_act_), which is then normalized to resting state (*S*_rest_) (Davis et al., 1998, Buxton et al., 2004), the Davis model predicts the following:

(1) $$\frac{\Delta S_{\mathrm{act}}}{S_{\mathrm{rest}}}\approx M\left[1-{\left(\frac{{CBF}_{\mathrm{act}}}{{CBF}_{\mathrm{rest}}}\right)}^{\alpha -\beta }{\left(\frac{{\mathrm{CMRO}}_{2\mathrm{act}}}{{\mathrm{CMRO}}_{2\mathrm{rest}}}\right)}^{\beta }\right]$$

where *a* is the Grubb's law exponent (Grubb et al., 1974) and *β* is a magnetic field-dependent parameter. *M* is a combined vascularization parameter that describes the variations that occur among different brain regions and across different time points and individuals. *M* can be theoretically derived:

(2) $$M=k\;TEV_{\mathrm{rest}}{\left(E_{\mathrm{rest}}B_{0}\right)}^{\beta }$$

where *k* is a proportionality constant, TE is the echo time, *V*_rest_ is the baseline blood volume, *E*_rest_ is the baseline oxygen extraction fraction, and *B*_0_ is the magnetic field strength.

The parameter *M* depends mainly on the baseline deoxyhemoglobin blood content, and thus it varies within subjects over time and within different brain regions and between subjects (Davis et al., 1998).

*M* can be directly estimated from hypercapnia calibration experiments, if it is assumed that changes are purely CO_2_ driven (i.e., *CMRO*_2***act***_ = *CMRO*_2***rest***_) without changes in neuronal activity as proposed by (Horvath et al., 1994, Yang and Krasney, 1995, Kastrup et al., 1999, Li et al., 1999, Kannurpatti et al., 2002, Cohen et al., 2004, Handwerker et al., 2007, Wise et al., 2013). Then Eq. (1) reduces to

(3) $$\frac{\Delta S_{{CO}_{2}}}{S_{\mathrm{rest}}}\approx M\left[1-{\left(\frac{CBF_{{CO}_{2}}}{CBF_{\mathrm{rest}}}\right)}^{\alpha -\beta }\right]$$

with $\Delta S_{{CO}_{2}}$ being the CO_2_-related signal change. A previous study (Kannurpatti and Biswal, 2008) has shown that the RSFA, estimated from rsfMRI data and conceptually identical to ALFF, is proportional to *M* and can be used as an alternative to hypercapnic experiments to determine $\Delta S_{{CO}_{2}}$.

Since RSFA and the task-related BOLD signal change are influenced by the same vascular physiology, *M* (Bandettini and Wong, 1997, Davis et al., 1998, Cohen et al., 2004, Kannurpatti and Biswal, 2008), a calibrated BOLD response amplitude *ΔS*_cal_ can be derived that is independent of inter-individual variations in *M* by dividing the tfMRI response amplitudes *ΔS*_act_ by RSFA $\Delta S_{{CO}_{2}}$ (i.e., Eq. 1 divided by Eq. 3):

(4) $$\Delta S_{\mathrm{cal}}=\frac{\Delta S_{\mathrm{act}}}{\Delta S_{{CO}_{2}}}=\;\frac{\left[1-{\left(\frac{{CBF}_{\mathrm{act}}}{{CBF}_{\mathrm{rest}}}\right)}^{\alpha -\beta }{\left(\frac{{\mathrm{CMRO}}_{2\mathrm{act}}}{{\mathrm{CMRO}}_{2\mathrm{rest}}}\right)}^{\beta }\right]}{\;\left[1-{\left(\frac{{CBF}_{{CO}_{2}}}{{CBF}_{\mathrm{rest}}}\right)}^{\alpha -\beta }\right]\;}$$

In most tfMRI experiments, it is not a single response amplitude *ΔS*_act_ that is estimated but rather multiple response amplitudes for different tasks or task components using a general linear model (GLM). Multiple task components are modeled with a set of regressors and result in the same number of estimated regression coefficients. Effects of interest are often summarized using contrasts, which are formed from a linear combination of the regression coefficients of the GLM. Contrasts can also be calibrated by dividing by $\Delta S_{{CO}_{2}}$ as shown in Eq. 4 since they are linearly related to the single responses. Assuming that the CO_2_-related signal change $\Delta S_{{CO}_{2}}$ and experimental effects (e.g., task responses) are additive, $\Delta S_{{CO}_{2}}$ can be directly estimated from the tfMRI data as the residuals of the GLM describing the experimental variance (i.e., the difference between the data and model prediction; see “Extracting $\Delta S_{{CO}_{2}}$ from the tfMRI data”).

### Methods

#### fMRI experiments

##### Data sets for validation of VasA fMRI

Since specialized data are not required by the proposed VasA fMRI approach, we used existing data from two different databases to assess its performance (the HCP and the PLORAS databases). The HCP data set (Barch et al., 2013, Glasser et al., 2013) included two sets of rsfMRI data (1200 frames per run; run duration 14 min 33 s) acquired on two separate days and tfMRI data sets for each participant (*n* = 80; age range: 22–35 years old; 58 are female, 19 are male, and 3 are not reported). Details about the tfMRI data sets used are provided in Table 1. Whole-brain multi-band single-shot echo planar imaging (EPI) acquisitions were acquired with a 32-channel RF receive head coil on a 3-T Siemens Skyra scanner with an enhanced gradient system. Imaging parameters were as follows: repetition time (TR) = 720 ms, echo time (TE) = 33.1 ms, flip angle = 52°, in-plane field of view (FOV) = 208 × 180 mm, 72 slices, 2 mm isotropic voxels, with a multi-band acceleration factor of 8. All HCP tasks and acquisition protocols are detailed in (Barch et al., 2013). Pre-processed HCP data were used (as described by Glasser et al., 2013, Parker Jones et al., 2014), ranging from 176 to 405 image volumes per time series depending on the task. The tfMRI data sets were acquired on two consecutive days at approximately 1:30 pm each day within approximately 30 min.

The PLORAS data set (Price et al., 2010) included tfMRI data (*n* = 58; 32 females, 26 males age range: 20–75; mean age 44). A two-dimensional single-shot EPI sequence was used with 3 × 3 mm in-plane resolution (TR/TE/flip angle = 3080 ms/30 ms/90°, FOV = 192 mm, matrix size = 64 × 64, 44 slices, slice thickness = 2 mm, inter-slice gap = 1 mm, 62 image volumes per time series). The PLORAS data were pre-processed using SPM12 (as described in (Hope et al., 2014)).

##### Rescaling of fMRI experiments

The HCP data sets were analyzed in three different ways: standard group statistics, rescaling using a rsfMRI reference data set, and the VasA fMRI approach. Since the PLORAS data sets did not include rsfMRI, they were only analyzed using standard statistics and VasA fMRI.

##### tfMRI data analysis for task-related activation maps

For the tfMRI data, we analyzed all available pre-processed data sets provided by the HCP (Glasser et al., 2013, Larson-Prior et al., 2013) and PLORAS (Price et al., 2010) studies using Statistical Parametric Mapping (SPM12; Wellcome Trust Centre for Neuroimaging, UCL, London, UK), implemented in MATLAB 7.14. Statistical analyses of the functional images were performed in two steps. Each subject's pre-processed tfMRI time series (unsmoothed data) underwent a fixed effects analysis, fitting the GLM at each voxel. Each event was convolved with a canonical hemodynamic response function. The data were high-pass filtered with a cutoff period of 128 s and were also corrected for serial autocorrelations using an autoregressive model. The contrasts of interest at the single-subject (first) level for each task were chosen as in (Barch et al., 2013) for the HCP data (Fig. 4). The contrasts of interest for the PLORAS data are presented in Fig. 4 (Hope et al., 2014).

##### Extracting $\Delta S_{CO_{2}}$ from the rsfMRI reference data

ALFF maps were computed for each subject using linearly detrended resting-state data (available only for HCP data sets). The ALFF map of the low-frequency power within the frequency band from 0.01 to 0.08 Hz was then estimated in each voxel for each subject and smoothed using an isotropic Gaussian smoothing kernel with 4 mm FWHM.

##### Extracting $\Delta S_{CO_{2}}$ from the tfMRI data using VasA

For tfMRI data (HCP and PLORAS), we used the residuals, i.e., the differences between the spatially unsmoothed tfMRI time series data and the GLM prediction. VasA low-frequency fluctuation maps were estimated from the residuals (after they were linearly detrended) in an analogous way to ALFF estimation (Zang et al., 2007). The time series for each voxel of the residuals map was Fourier transformed and the power spectrum was obtained. The averaged square root of the power within the frequency band of 0.01–0.08 Hz was then calculated at each voxel. The resulting low-frequency fluctuation maps were smoothed with an isotropic Gaussian kernel with 4 mm FWHM.

##### Rescaling the BOLD response and analysis of contrasts

For each individual, a contrast image describing the activation in each task (Fig. 4) was created. The contrast image was then smoothed using an isotropic Gaussian kernel with 4 mm FWHM and was either not calibrated (standard approach) or calibrated, i.e., the voxel-wise contrast estimate was divided by $\Delta S_{CO_{2}}$, i.e., the respective ALFF or VasA residual estimates. The standard or rescaled contrasts were then entered into a second-level analysis (i.e., uniform effect analysis using a summary statistic approach) to enable inferences at the group level. *T*-statistics were estimated for each contrast at the group level and thresholded at *p* < 0.05 (with family-wise error (FWE) correction for multiple comparisons). The *t*-scores (for the positive contrast) from either ALFF calibration or VasA fMRI were plotted against the *t*-scores from the standard analysis for all voxels that survived FWE correction with *p* < 0.05. The percentage change in *t*-score due to rescaling was derived from a linear regression of these scatter plots.

##### Determining false-positive rates of VasA-based fMRI analyses

Ten thousand simulations were performed, each of them using the 80 subjects from the HCP data for an arbitrarily selected motor mapping task. For each simulation, a subject-specific random regressor was added to each design matrix at the first level. The random regressor was formed by convolving normally distributed noise, Ɲ (0,1), with the canonical hemodynamic response function (HRF) implemented in SPM12. The convolution with the HRF was performed to closely mimic typical BOLD responses and simulate the covariance structure of fMRI data. A contrast testing for the explanatory power of the random regressor, which should be zero, was specified for every subject and the null hypothesis that its population mean is zero was tested at the group level using *F*-statistics with *p* < 0.05 (FWE corrected).

##### Optimal frequency band for estimating fluctuations relevant for rescaling

VasA maps were computed using a range of different frequency bands by varying the upper limit from 0.02 to 1.2 Hz in increments of 0.01 Hz (whilst fixing the lower frequency to 0.01 Hz). Normalization of the BOLD response and the analysis of the contrasts were performed as described above in a motor task from the HCP data set (Table 1). The percentage increase in the number of activated voxels was then used as a measure of improved sensitivity for each tested frequency band.

##### Comparison of VasA fMRI to CBV VASO and CVR maps

In order to elucidate the physiological underpinnings of the VasA method, VasA maps from a visual checkerboard stimulation experiment were compared to relative CBV estimates from VAscular Space Occupancy (VASO) acquisitions (experiment 1, *n* = 3 volunteers) and CVR estimates (experiment 2, *n* = 1 volunteer). Both experiments were performed at 7 T at the Max Planck Institute for Human Cognitive and Brain Sciences, Leipzig.

##### Experiment 1: CBV VASO

The applied fMRI sequence consisted of an interleaved acquisition scheme of combined T_1_ and T_2_* weighting providing a CBV weighted VASO contrast in addition to the BOLD signals at rest by combining a T_1_ preparation module with a multi-echo readout (Huber et al., 2014). tfMRI data were acquired on a Siemens MAGNETOM 7 T scanner (Siemens Healthcare, Erlangen, Germany) in 3 subjects (2 female, age = 22–25 years). For RF transmission and reception, a 24-channel receive and a circularly polarized single-channel transmit head coil (Nova Medical, Wilmington MA, USA) were used. In a 10-min long fMRI run a visual checkerboard (8Hz) stimulus (30 s rest vs. 30 s stimulation) was used to activate the visual cortex. Data were acquired in five axial slices aligned along the calcarine sulcus with a two-dimensional single-shot gradient-echo EPI readout. Due to small head motion, parts of the two outermost slices were disregarded in the analysis. The imaging parameters were TE/TI/TR = 16/1000/1500 ms, nominal voxel size of 2 × 2 × 2 mm^3^, partial Fourier factor 6/8 in the phase encoding (PE) direction.

These data were receive-field corrected, and low-frequency fluctuation maps of the low-frequency power within the frequency band from 0.01 to 0.08 Hz were extracted from VASO data (TR = 1500 ms, 255 volumes) acquired at rest (normalized with respect to the mean signal to reflect %CBV changes). The VasA maps were extracted from the residuals after applying the GLM from BOLD checkerboard tfMRI data in the same subjects (using the exact procedure described above for the PLORAS/HCP data), and correlation tests were performed between the VasA maps and the CBV maps (*n* = 3). The voxel-wise correlation between VasA and CBV maps was estimated across all gray matter voxels with a significance threshold *p* < 0.05.

##### Experiment 2: CVR

A single subject (female, age 31 years) was scanned during a 12-min long hypercapnia task consisting of 2/5/5 min of breathing air/5% CO_2_, 21% O_2,_ and balanced N_2_/air. The pre-mixed gas composition was delivered via a non-rebreathing mouthpiece connected to a three-way valve separating the inflow gas from the gas exhaled by the subject. The heart rate and the respiratory gas composition were recorded with a BIOPAC MP150 unit (BIOPAC Systems Inc, Goleta CA, USA). Inhaled and exhaled air samples were continuously taken via a small flexible tube attached to the participant's mouthpiece and connected directly to the gas sensor of the O_2_ and CO_2_ modules of the BIOPAC system. The BIOPAC system was calibrated before the experiment by adjusting the input resistances for both module sensors to the known partial pressures of two gas mixtures. For comparison with neural activity induced signal changes and testing VasA, a 10-min flickering checkerboard (8 Hz) stimulation experiment (alternating 30 s rest vs. 30 s stimulation) (same as that performed in Experiment 1) was conducted right after the hypercapnia experiments.

To account for the prominent decrease of arterial arrival time during hypercapnia and to avoid inflow effects of fresh (non-inverted) blood in VASO, the blood-nulling time was reduced to TI = 765 ms by adjusting the adiabatic inversion pulse efficiency to 75% in a B_1_-independent way (Huber et al., 2014).

A medical doctor was present in the magnet room at all times during the breathing manipulation and was responsible for adjusting gas flow rates and monitoring heart and breathing cycles. All procedures of the experiments with breathing manipulations were approved by the Ethics Committee of the University of Leipzig, and informed written consent was given by the participant.

The CVR data were motion corrected and CVR maps were calculated voxel by voxel in units of % signal change per mmHg end-tidal CO_2_ change. fMRI signal and end-tidal CO_2_ changes were averaged over the period of 3 min. The first 2 min of breathing 5% CO_2_ were excluded from the analysis to minimize effects of signal transition between steady-state rest steady-state hypercapnia.

The pulse sequence used for the CVR experiment was the same as for experiment 1 but with different imaging parameters: TE/TI/TR = 19/765/1500 ms, nominal voxel size of 1.5 × 1.5 × 1.5 mm^3^, partial Fourier factor 5/8 in the PE direction. The VasA maps were extracted from the residuals after applying the GLM to the BOLD checkerboard tfMRI data as above. Correlation tests were performed between the VasA map and the CVR map (*n* = 1). The voxel-wise correlation between VasA and CVR maps was estimated across all gray matter voxels with a significance threshold of *p* < 0.05.

## Results

**Fig. 1:**
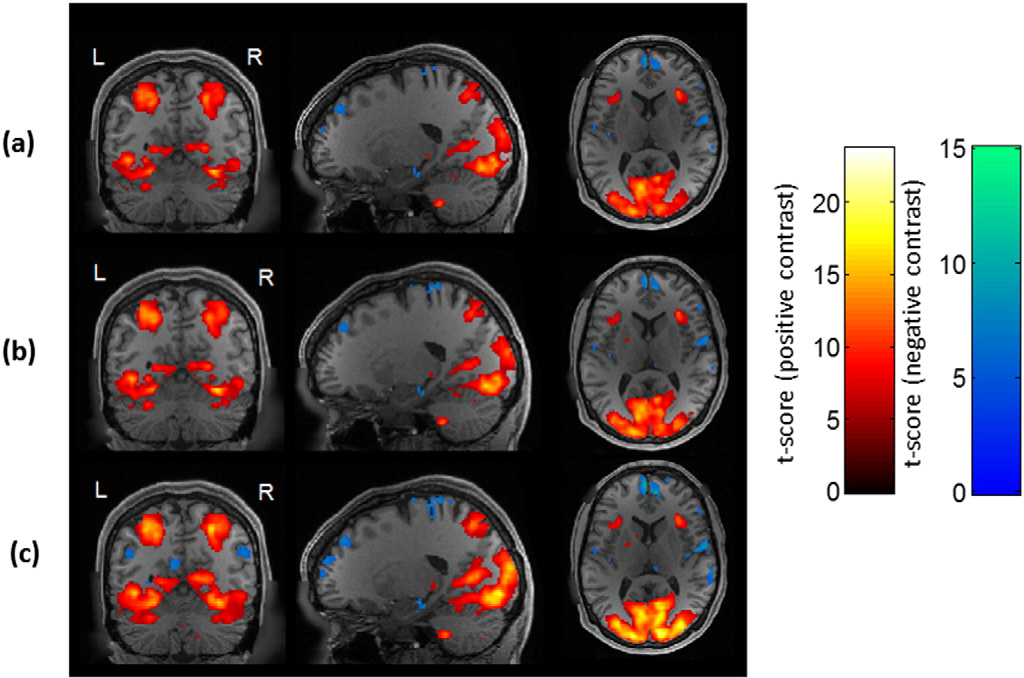
Group-level activation maps (*p* < 0.05, FWE) for a relational processing task (contrast: relational processing task versus baseline) using the standard analysis (a), using rescaling with a rsfMRI reference scan (b) and VasA fMRI (c). In the relational processing task, participants were presented with 2 pairs of objects with one pair at the top of the screen and the other pair at the bottom of the screen. They were then asked to decide what dimension differs across the top pair of objects and whether the bottom pair of objects differs along the same dimension (Barch et al., 2013). The contrast reflects the difference between the relational processing relative to the baseline condition.

**Fig. 2:**
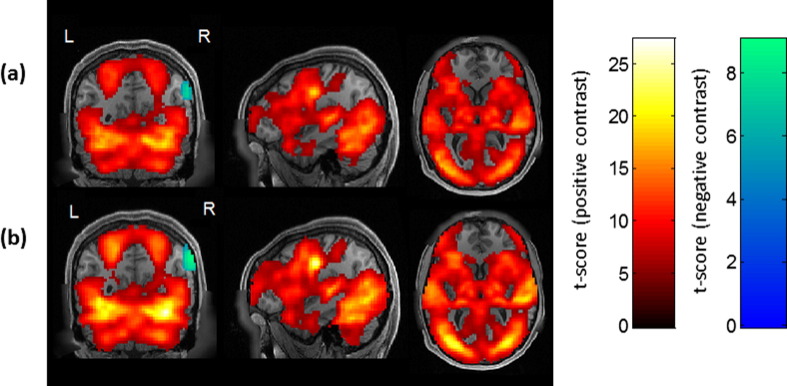
Group-level activation maps (*p* < 0.05, FWE) for a speech-processing task using the standard analysis (a) and VasA fMRI (b). In the speech-processing task, volunteers were instructed to say one word. The contrast reflects the difference between the speech processing (reading) relative to the baseline condition (marker fixation).

**Fig. 3:**
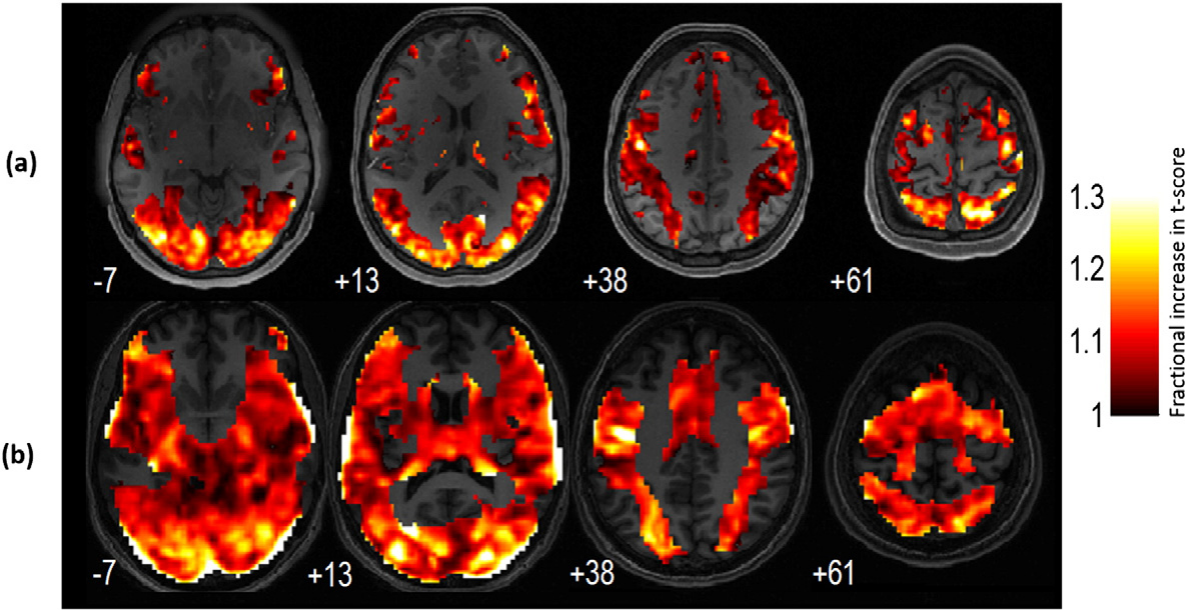
Average functional sensitivity improvement using VasA for all brain areas that were tested using all available tfMRI data from the HCP (19 contrasts) (a) and PLORAS data (14 contrasts) (b). Sensitivity increases exceeded 30% in several brain areas, e.g., the occipital cortex or the temporal pole, and were consistent across the different data sets. Note that negative values are not shown because only 3% of all activated voxels showed a minor reduction of less than 2%. The color bar represents fractional increases in *t*-score.

**Fig. 4:**
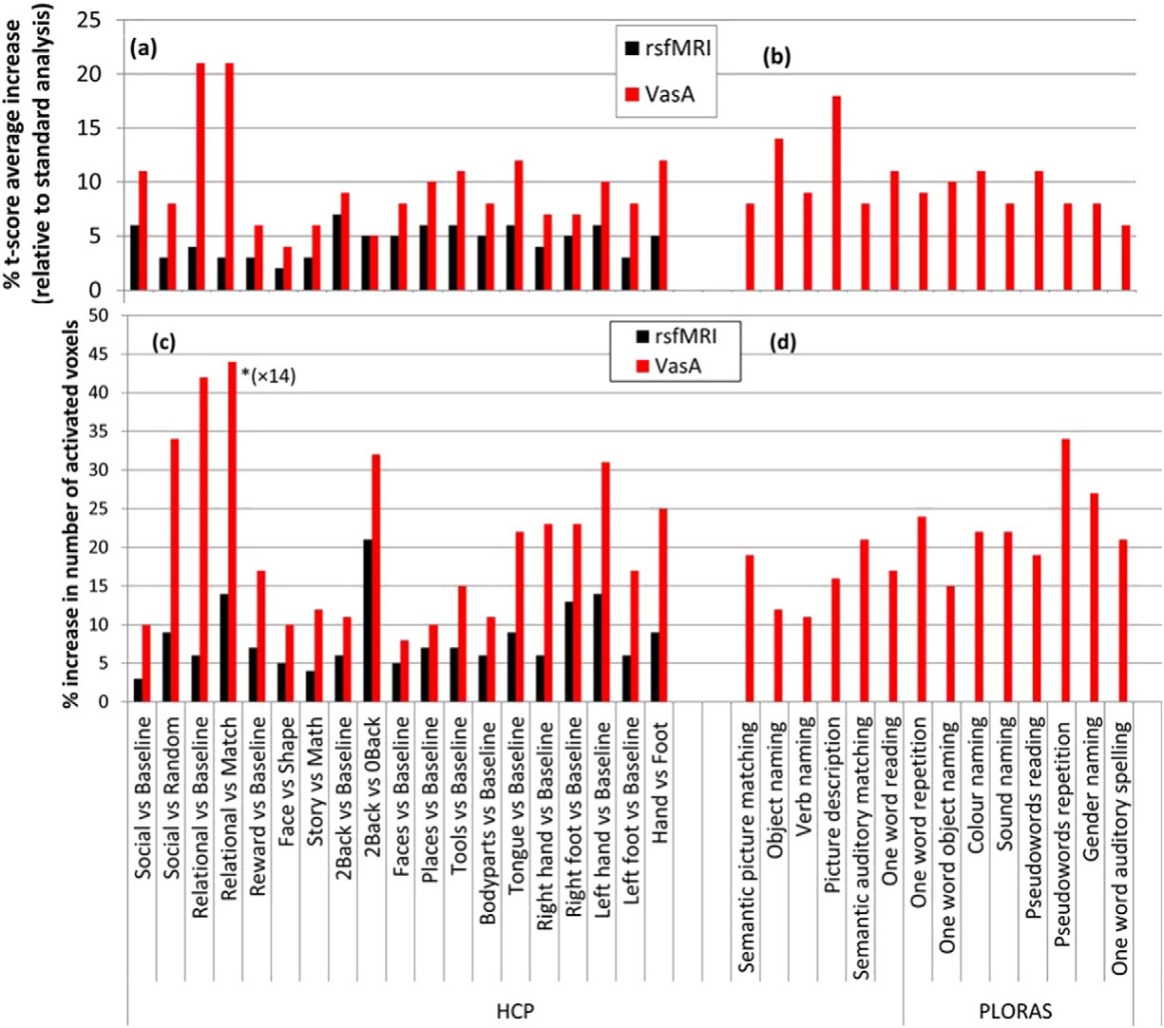
Mean sensitivity increases due to the rsfMRI and VasA fMRI rescaling methods compared to the standard approach. Bars indicate the relative percent increase in average *t*-score for HCP (a) and PLORAS (b) data sets and the relative increase in number of activated voxels (*p* < 0.05, FWE) for specific contrasts of the HCP (c) and the PLORAS (d) data sets. Increases were averaged across the entire brain, i.e., local sensitivity increases were considerably higher in many brain areas (see Fig. 1, Fig. 2, Fig. 3). Note that the axis was shortened for the relational processing task (*) in panel c; the total number of activated voxels increased from 159 voxels to 1139 voxels when using VasA instead of the standard analysis, resulting in 616% increase in the number of activated voxels for this task.

VasA fMRI increased the functional sensitivity substantially for a range of different experiments and data acquisition schemes (Fig. 1, Fig. 2, Fig. 3, Fig. 4). On average ,the mean *t*-score increase across all activated brain areas was approximately 10% but it reached up to 21% for particular experiments (e.g., relational processing experiment in Fig. 4). VasA fMRI yielded considerably higher *t*-scores (approximately 130% higher) than the rescaling based on rsfMRI data (Fig. 4). Fig. 1 shows an example of a group-level statistical map for 80 subjects for a relational processing task from the HCP study (Barch et al., 2013) analyzed with the standard and two different rescaling approaches. Fig. 2 shows another example of activations in 58 volunteers performing a speech-processing task from the PLORAS study (Price et al., 2010). In general, the rescaling methods, particularly VasA fMRI, increased the spatial extent of activations and *t*-score values across the entire brain. In some areas, the *t*-score increases relative to the standard analysis exceeded 30%, such as in the visual cortex (Fig. 1c). As can be seen in Fig. 1, Fig. 2, Fig. 4 similar increases were achieved for different types of data sets, i.e., the increased sensitivity afforded by VasA fMRI was independent of task and data acquisition.

### Local sensitivity increases

Fig. 3 and movie (supplementary material) summarizes the sensitivity improvements that were achieved in different brain areas using VasA fMRI. It shows the mean *t*-score value increases due to VasA in each voxel derived from all fMRI data from HCP (a) and PLORAS (b) data, i.e., it pools over all tasks.

### Spatial correlation between the resting-state ALFF and VasA maps

**Fig. 5:**
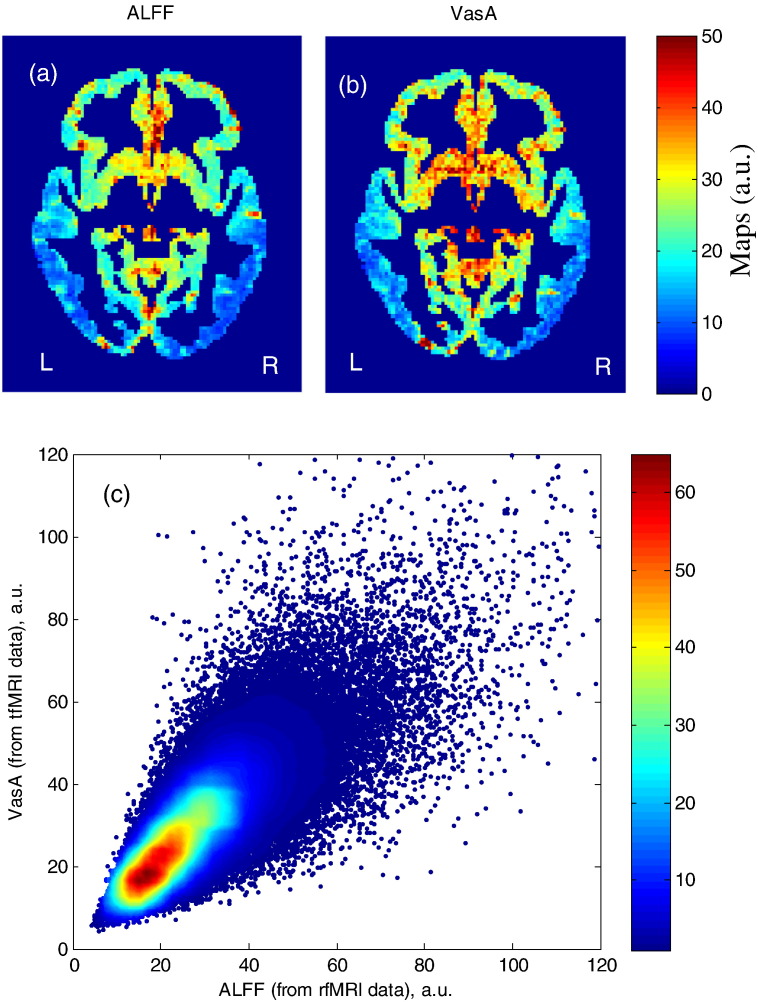
Comparison between ALFF (derived from rsfMRI data, a) and VasA maps (derived from a working memory task, b) for the same single subject; voxel-wise scatter density plot (of the data shown in panels a and b) between unsmoothed ALFF (derived from rsfMRI data) and unsmoothed VasA maps (derived from tfMRI HCP data (working memory), measured in arbitrary units (a.u.)) (c). A gray matter tissue mask was applied.

**Fig. 6:**
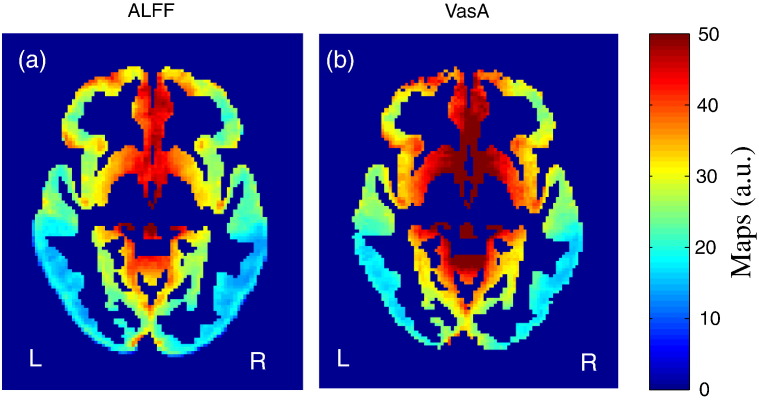
Group average ALFF (derived from rsfMRI data) and VasA maps (*n* = 80, derived from tfMRI HCP data (working memory), measured in same arbitrary units (a.u.)).

There was a strong correlation between ALFF and VasA measures (Spearman's rank correlation coefficient *r* = 0.78 for *n* = 80 subjects unsmoothed). Figs. 5(a) and (b) show a single subject comparison between ALFF and VasA maps. Fig. 5(c) shows a scatter density plot for a representative subject (unsmoothed maps measured in arbitrary units (a.u.)). Fig. 6 shows a group comparison between ALFF (extracted from a resting-state experiment) and VasA (extracted from tfMRI HCP data, working memory task) maps (*n* = 80 subjects).

### Control of false positives

Permutation analysis demonstrated that the rate of false-positive activations was adequately controlled by VasA fMRI. It was based on adding and testing an extra randomly generated regressor to the original tfMRI general linear model, which should not significantly explain any time series variance. Simulation testing was repeated for *n* = 10000 permutations and resulted in false-positive activation rates of less than the targeted 5%, i.e., 2.14% (95% confidence interval: [0.0187, 0.0244]).

### Optimal frequency band for estimating fluctuations relevant for rescaling

**Fig. 7:**
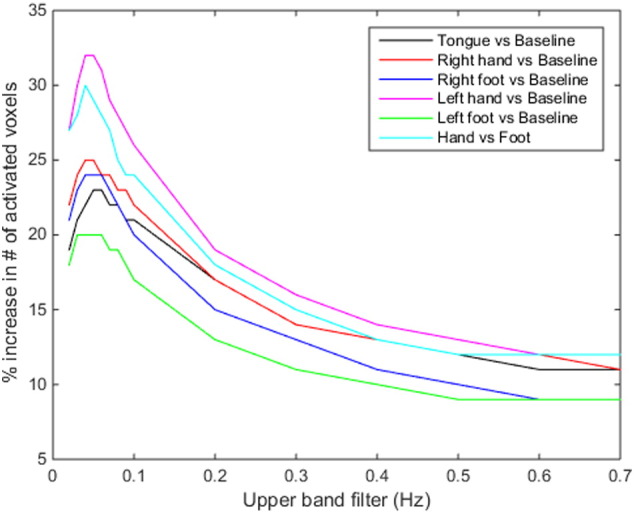
Effect of the choice of the frequency band used for determining the VasA vascular estimate. The upper cutoff of the band-pass filter was systematically varied and the improvement due to VasA quantified as the increase in the number of activated voxels across the brain. The data were taken from different motor tasks from the HCP data set.

The maximum sensitivity improvements were achieved by extracting the CO_2_-driven low-frequency fluctuations from a frequency band with a low cutoff at 0.01 Hz and high cutoff at 0.1 Hz. The improvements were stable over high cutoff frequencies ranging from 0.06 Hz to 0.1 Hz (average variation in number of activated voxels was 24%) for a randomly selected HCP motor mapping task data set (Fig. 7). When higher frequencies for the high cutoff were used (> 0.1 Hz), the improvement was substantially lower (12%), indicating that the band-pass filter used for VasA (0.01 Hz–0.08 Hz) captured the vascularization differences effectively.

### Comparison of VasA maps to CBV VASO and CVR maps

**Fig. 8:**
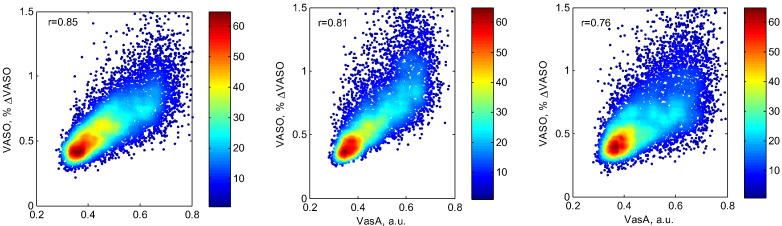
Correlation between low-frequency %CBV fluctuation maps extracted from the VASO data (normalized with respect to the entire frequency band to reflect CBV changes) at rest and the corresponding maps extracted from the residuals after applying the GLM from BOLD tfMRI data (checker board experiment) in the three subjects at 7 T (*r* = 0.85/0.81/0.76 for subjects 1–3, respectively)).

**Fig. 9:**
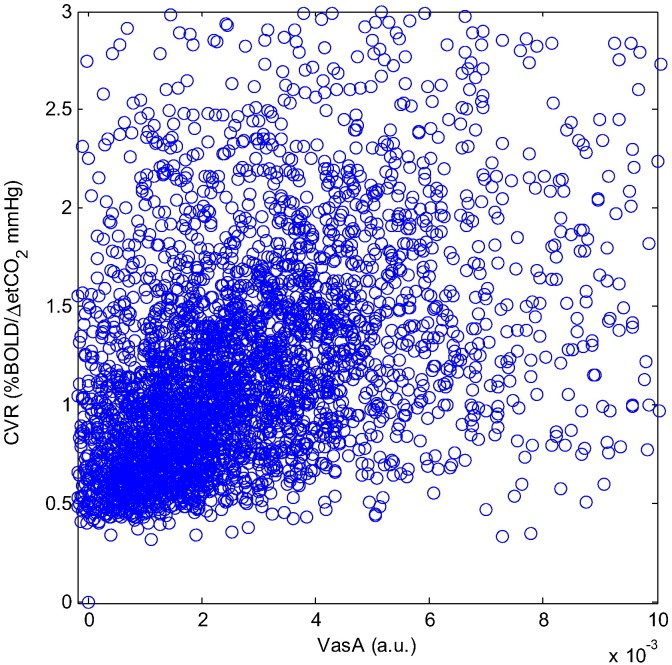
Scatter plot comparing VasA and CVR measures across the brain gray matter (*r* = 0.39).

There was a strong positive correlation between the VASO low-frequency %CBV maps and the corresponding VasA maps (Fig. 8; all *r* > 0.76, all *p* < 0.05) in all three subjects. The CVR measures were significantly correlated with the VasA measures in the single subject (Fig. 9, *r* = 0.39, *p* < 0.05).

The higher sensitivity improvement due to VasA fMRI compared to rsfMRI-based calibration was further investigated by quantifying the reproducibility of ALFF maps across different time points. The coefficient of variation (CoV) of ALFF maps was significantly smaller when they were estimated from the same experimental run as done for VasA rather than from separate experiments as in rsfMRI calibration. The CoV of ALFF maps between two different runs (acquired on two consecutive days) was 14 ± 3% and within one run was 11 ± 2%. That is, the CoV of ALFF maps was significantly reduced by 21% when it was estimated from the same experimental run as opposed to when it was estimated from two different runs (Student's paired *t*-test, *p* < 0.0001; *t* = − 8.86).

### Improved sensitivity in areas with low SNR

VasA fMRI also improves functional sensitivity in areas which are known to have very low-functional sensitivity and suffer from signal dropout (Table 2). For example, the percentage increase in the number of activated voxels relative to the standard approach was 66% in ventral prefrontal cortex, 31% in the amygdala, 29% in hippocampus, and 200% in the orbital frontal cortex.

The improved sensitivity can be leveraged in several ways. The detection power of studies may be considerably increased or group sizes may be reduced while retaining the same power. A *t*-score increase of 20% may translate into a reduction of approximately 40% in the group size while achieving the same statistical power, assuming simple Gaussian noise.

## Discussion

Functional MRI studies aim to infer the neuronal activity from the measured BOLD response. Thus, any non-neuronal contributions or modulations of the signal reduce the sensitivity and introduce bias. We developed a principled rescaling technique, VasA fMRI that reduces inter-individual vascularization variations that modulate the BOLD signal and significantly contribute to the inter-subject variance in fMRI group studies. Compared to standard analysis, VasA fMRI significantly increases the sensitivity of group studies in terms of *t*-score values by up to 30% in specific brain areas without increasing the false-positive rate. For reference, a 20% increase is comparable to sensitivity improvements due to increasing the field strength from 1.5 T to 4 T for group studies (Yang et al., 1999).

### Relation to BOLD physiology

VasA fMRI estimates a measure of local vascularization directly from the time series data of an fMRI experiment. The residual variance after fitting the experimental model yields an estimate of the spontaneous low-frequency fluctuations in the BOLD signal. These fluctuations are hypothesized to relate to fluctuations in the arterial CO_2_ level and subject-specific vascularization differences such as the local blood flow/volume (Wise et al., 2004). A principled derivation (see Theory section) shows the relationship between this vascularization measure and the established vascularization parameter (*M*) in the Davis model (Davis et al., 1998), which modulates the BOLD signal multiplicatively. Thus, the simple scaling used in VasA fMRI can address the inter-individual differences.

### Comparison with reference scan-based rescaling methods

Compared to rescaling methods using separate reference scans such as the ALFF method based on rsfMRI data (Kannurpatti and Biswal, 2008), the performance of VasA fMRI is higher (i.e., we observed consistently higher *t*-score values and more activated voxels for VasA in comparison to scaling using the rsfMRI-based ALFF measure). One important reason may be that in VasA fMRI, the rescaling is derived from the same time series as the tfMRI. Hence, it may more closely reflect the current physiological state during the task than rsfMRI reference scans acquired in another session. We note that VasA relies on the standard time series GLM-based statistical analysis and thus differs from a recent rescaling method by (Kannurpatti and Biswal, 2008), where the noise estimates for the statistics were determined from the variations in percentage signal changes across the brain (but not a time series). The method of (Kannurpatti and Biswal, 2008) may be used for analysis of single scans and subjects, whereas VasA is tailored towards multi-subject group analyses.

Unlike other correction methods, VasA fMRI can be applied retrospectively to all existing task fMRI experiments because it does not require additional and complicated reference scans. Skipping the reference scans not only saves time but is also more comfortable for the subject. For example, in the established hypercapnia calibration approach, volunteers need to breath CO_2_ enriched air (Davis et al., 1998), which can induce adverse breathlessness, potential sensory stimulation, and is not practical for many neurological and psychiatric conditions. As an alternative to breathing CO_2_ enriched air, breath-hold can be used to induce mild hypercapnia (Kastrup et al., 1999, Thomason et al., 2005, Handwerker et al., 2007). However, this method also induces stress in the subject and has poor reproducibility (Chiarelli et al., 2007). Moreover, all approaches assume that brain metabolism is not affected (Kannurpatti and Biswal, 2008, Zappe et al., 2008, Mohtasib et al., 2012), which is controversial (Mohtasib et al., 2012). Inducing hyperoxia with O_2_ enriched air instead of hypercapnia is considered more tolerable (Chiarelli et al., 2007) but faces similar concerns regarding its validity (Hoge, 2012).

### Considerations

The improvement in functional sensitivity due to VasA fMRI varies across brain areas (Fig. 3). We hypothesize that this variation may be due to different factors: (1) residual inter-individual anatomical differences may have remained even after spatial normalization and affected different regions to varying degrees; (2) the different tasks tested here induce activations in different brain areas and, thus, inter-individual differences in task performance may have resulted in higher variation of neuronal and consequent BOLD responses for some areas over others; (3) task-correlated changes in breathing patterns where in some complex tasks, both cognitive and motor subjects may hold their breath or increase their breathing rate during the task; and (4) individuals may vary greatly in their resting CO_2_ level, and in how this changes during breathing and deliberate manipulations of inspired gases (Prisman et al., 2008). This may also account for some small differences between the results for the HCP and PLORAS data (Fig. 3a vs. b). Moreover, VasA does not capture all variance components expressed in the vascularization parameter *M* (Eq.2).

Importantly, the sensitivity improvements were found consistent across very different acquisition protocols (with e.g., repetition time TR = 0.7 s vs TR = 3 s; Fig. 3) and field strengths. As the VasA principle was derived from fundamental BOLD signal models, it is expected that it can be applied at other field strengths as well.

VasA accounts for differences in the calibration parameter *M*, which depends on baseline blood volume and baseline oxygen extraction fraction (Eq. 2) and differs between subjects. VasA does not fully account for baseline CBF differences since a CBF dependence remains even after autorescaling, Eq. 4. Thus, it may not account for all sources of vascular variance across subjects. The inter-subject variability of *M* is much higher (Table 1 of (Davis et al., 1998), coefficient of variation (CoV) of *M* = 0.30) than the inter-subject variability in the ratio of CBF during hypercapnia to baseline ($\frac{{CBF}_{{CO}_{2}}}{{CBF}_{\mathrm{rest}}}\text{,}$ which has CoV of 0.06). The CoV of *M* is also much higher than the ratio of CBF during stimulation to baseline ($\frac{{CBF}_{\mathrm{act}}}{{CBF}_{\mathrm{rest}}}\text{,}$ which has CoV of 0.08) (Davis et al., 1998). Thus, we expect VasA to account for the majority of vascular differences since it removes sensitivity to inter-subject variance in *M*.

VasA fMRI is based on the assumption that variations in CO_2_ have no effect on the cerebral metabolic rate. Debates concerning this hypothesis have been prevalent in the field for the last 30 years (Siesjo, 1980, Yablonskiy, 2011); with some reporting increases (Horvath et al., 1994, Jones et al., 2005), decreases (Zappe et al., 2008, Xu et al., 2011) and even no changes in CMRO_2_ (McPherson et al., 1991, Hino et al., 2000, Chen and Pike, 2010). Although this leads many to believe that this disparity in results arises due to differences in experimental methodology, one relationship is clear: the level of the CMRO_2_ changes is related to the severity of the CO_2_ change. Direct electrophysiological recordings from rodent brain show clear changes in baseline neuronal activity during severe 10–12% hypercapnia (Jones et al., 2005, Kennerley et al., 2012) but negligible changes for smaller < 5% CO_2_ variations (Jones et al., 2005). VasA calibration relies on minor variations in CO_2_ caused by natural breathing changes that are expected to be smaller than CO_2_ variations arising from breathing manipulation. Therefore, one can reasonably presume variations in CMRO_2_ are negligible under our experimental conditions.

While VasA is believed to generally well correct for inter-regional and inter-subject variations in vascular reactivity, it cannot account for all sources of physiological variability. Physiological factors that could affect VasA include vasomotion, which is a local phenomenon that relates to the oscillation of the vascular diameter (i.e., rhythmic change in diameter) at frequencies in the range from 1 to 20 min^- 1^ (Slaaf et al., 1988, Mayhew et al., 1996, Aalkjaer and Nilsson, 2005). The origin of vasomotion and its physiological consequences are still not completely understood (Nilsson and Aalkjaer, 2003). Particularly, the links between neural activity and vasomotion are still unclear (Sirotin and Das, 2009) with some papers reporting that it is not related to respiration, heartbeat, or neuronal input (Schechner and Braverman, 1992, Porret et al., 1995, Aalkjaer and Nilsson, 2005). The band of frequencies that are related to vasomotion in small arteries are believed to be between 0.02 and 0.2 Hz (Aalkjaer and Nilsson, 2005) combination of harmonics and slower oscillatory envelopes. As high-pass filtering is a standard preprocessing step in the fMRI data analysis, this will remove any vasomotion-related harmonics above the typical cutoff of 0.1 Hz, significantly reducing any potential impact of vasomotion. VasA will not be able to distinguish between slow oscillations related to vasomotion or CO_2_ fluctuations, which VasA targets. Adding to the complexity, the two effects could be linked and further research is required to untangle the two. Thus, it cannot be excluded that vasomotion effects affect VasA estimates and their interpretation to a certain degree.

Changes in heart rate are another physiological factor that may affect VasA (Shmueli et al., 2007, Chang et al., 2009). Shmueli et al. (2007) reported that heart rate fluctuations explain 1% of resting-state BOLD signal variance. This variance was not concentrated entirely around large cerebral blood vessels but also included gray matter. The authors reported a complex temporal relationship between heart rate and the BOLD signal; the heart rate was negatively correlated with the BOLD signal amplitude at time lags ranging from 6 to 12 s, and positively correlated at time lags of 30–42 s. The heart rate fluctuations may therefore bias VasA estimates.

VasA fMRI relies on an accurate model of the experimental task-induced variance; an inaccurate or incomplete model will result in neuronal confounds. In this case, the VasA estimates may be inaccurate and lead to suppression of real activation. If the model is inadequate or incomplete, the vascular estimate may be inaccurate and lead to suppression of real activation. The mis-specification of the model may result from simple experimental issues such as stimulus timing errors but also from neuronal resting-state activity that is not modeled. Potential task-related respiratory changes may not be properly captured by the model either. Any mis-specification will increase the residuals of the GLM fit. Since VasA uses the power of the residuals in a specific low-frequency band, it is rather robust against mis-specifications. In the less likely case that the residuals have a significant component in the VasA frequency band, they will always result in a spurious increase of the VasA estimate (but no decrease). It is plausible that for most cases of mis-specifications the error will be similar across subjects (e.g., for a general timing error). In this case, the percent increase in the VasA estimate will be similar across subjects and thus the relative scaling between subjects and brain areas will be preserved. Thus, VasA is expected to achieve the same relative improvement in sensitivity in this case.

In the less likely case that the mis-specifications are subject dependent, reductions in sensitivity are in principle conceivable. However, in these cases, it is likely that the entire experiment and standard analysis approach will be generally affected. If such a case is suspected, the data can additionally be analyzed with the standard approach since VasA is a post-processing method and does not require tailored data acquisition.

Susceptibility artifacts may potentially influence the VasA estimation. The magnitude and extent of susceptibility artifacts in basal brain areas will change with respiration (Menon et al., 1993, Deichmann et al., 2002, Weiskopf et al., 2006). These signal changes may be picked up by the VasA scaling procedure. Future studies using recordings of peripheral physiology and directly measuring dynamic susceptibility effects will help quantifying this effect (Hutton et al., 2008).

Although VasA may theoretically reduce the sensitivity, our study and results support that this case is highly unlikely. Unlike previous studies (Kannurpatti and Biswal, 2008, Kalcher et al., 2013), we applied VasA fMRI to a very wide spectrum of tasks and data sets (Table 1) and the method in every single case outperformed the existing methods (i.e., higher t values and increased number of activated voxels). Although we successfully applied VasA to a wide range of fMRI paradigms and a large number of volunteers, we note that it is not completely clear how well the method will generalize to any arbitrary task or pathophysiological cases. Thus, we cannot fully exclude the possibility that VasA may underperform and reduce functional sensitivity in these particular cases.

### Improved sensitivity and reduced bias

VasA fMRI supports the detection of subtle group effects. For several brain areas, the increase in sensitivity is comparable to the increase observed for an increase in field strength from 1.5 T to 4.0 T or the use of multi-channel RF head coils instead of single-channel coils (ca. 20% *t*-score increase (Yang et al., 1999)).

Vascularization may systematically vary between groups, e.g., for different age-groups (Richter and Richter, 2003) or patient versus control groups. Thus, the group analyses might not reflect differences in neural activity but rather difference in vascular responses (Thomason et al., 2005). For such studies, VasA fMRI may capture some of the systematic differences. Although this study did not demonstrate it, VasA promises a simple practical way of reducing the probability of misinterpreting spurious vascular group differences as neural in origin to a certain degree (Chugani et al., 1987, Gaillard et al., 2001).

### Physiological basis of the VasA maps

The VasA estimates were correlated with CVR and VASO CBV measurements, in line with the assertion that VasA adjusts for inter-individual vascular response differences. The VasA maps correlate more strongly with CBV-based VASO signal fluctuations (in units of %ΔVASO) acquired during resting state, suggesting that a large portion of the VasA maps reflects baseline vascularization. The observed correlation with CVR maps may be driven by the relationship to CBV since CVR generally depends on CBV (Ainslie and Duffin, 2009). We note that not all sources of physiological variations could be explained.

The use of VasA maps as a vascular scaling factor is not only supported by our data but also by recent work by Tsvetanov et al. (2015), where the authors showed evidence from magnetoencephalographic recordings (MEG) of how scaling by RSFA could be used to separate neuronal from vascular confounds in an ageing study. The authors used mediation models to determine the variability differences between the vascular and the neural mediators for effects of age on RSFA. They reported that 48% of the variance is explained by the vascular mediator in comparison with 7% explained by the neural mediator (Tsvetanov et al., 2015).

To more comprehensively understand and robustly assess the physiological underpinnings of VasA, follow-up studies will need to consider the other components influencing the BOLD response such as CBF, CBV, and CMRO_2_ (Blockley et al., 2013).

## Conclusion

VasA fMRI significantly increases functional sensitivity in group analyses. It is straightforward to add to current analysis methods and can be applied to any task fMRI data set irrespective of the task or acquisition protocol. It does not require additional reference scans or other complicated procedures. In our study, VasA clearly outperformed the standard analysis and a current alternative rescaling approach based on rsfMRI data. VasA fMRI efficiently improves sensitivity. If BOLD amplitude rescaling is desired, VasA reduces scanning time and stress for patients compared to existing calibration methods. While future studies will be required to further determine the detailed mechanisms behind the VasA approach, the explorative comparisons between VasA and other BOLD parameters, including CVR and CBV, suggest that VasA is generalizable to a broad set of tasks and experimental conditions.

## Figures and Tables

**Table 1:** tfMRI HCP data (Barch et al., 2013).^a^

Task Day of the task^b^	No. of task blocks in each run	Frames per run	Run duration (min:s)	Contrast used
Social cognition Theory of mind (Day 2)	5 (50% of blocks TOM,^c^ 50% of blocks random)	274	3:27	Social vs baseline Social vs random
Relational processing (Day 2)	6 (50% of task blocks relational, 50% of task blocks control)	232	2:56	Relational vs baseline Relational vs match
Gambling (Day 1)	4 (50% of task blocks reward, 50% of task blocks punish)	253	3:12	Reward vs baseline
Emotion processing (Day 2)	6 (50% of task blocks face, 50% of task blocks shape)	176	2:16	Face vs shape
Language (Day 2)	8 (50% of task blocks story, 50% of task blocks math)	316	3:57	Story vs math
Working memory (N-Back Task) (Day 1)	8 (50% of task blocks 0-back, 50% of task blocks 2-back)	405	5:01	2Back vs baseline 2Back vs 0-back Faces vs baseline Places vs baseline Body parts vs baseline
Motor (Day 1)	10 (20% of for each body part (5 parts in total)^o^)	284	3:34	Tongue vs baseline Right hand vs baseline Right foot vs baseline Left hand vs Baseline Left foot vs baseline Hand vs foot

^o^ body parts included: Right hand, left hand, right foot, left foot, tongue.

a Adapted from Barch et al. (2013).

b Tasks were carried out in two consecutive days (Day 1 and Day 2)

c Theory of mind.

**Table 2:** Percent increase in the number of activated voxels (*p* < 0.05, FWE) using VasA fMRI compared to the standard approach. Data were from the HCP study and increases were measured across different regions of interest (ROI).

ROI	% Increase in number of activated voxels
Anterior cingulate	70
Inferior frontal gyrus	12
Superior temporal gyrus	9
Lateral occipital	54
Superior frontal	28
Insular	10
Middle frontal gyrus	25
SMA	14
Superior parietal	11
Hippocampus	29
VMPFC	66
Amygdala	31
Visual cortex	21
Temporal (sup and inf)	26
Orbital frontal	200
Pallidium putamen	69
Motor	10

## References

[bb0005] Aalkjaer C., Nilsson H. (2005). Vasomotion: cellular background for the oscillator and for the synchronization of smooth muscle cells. Br. J. Pharmacol..

[bb0010] Ainslie P.N., Duffin J. (2009). Integration of cerebrovascular CO_2_ reactivity and chemoreflex control of breathing: mechanisms of regulation, measurement, and interpretation. Am. J. Physiol. Regul. Integr. Comp. Physiol..

[bb0015] Ashburner J., Friston K.J. (2011). Diffeomorphic registration using geodesic shooting and Gauss–Newton optimisation. NeuroImage.

[bb0020] Bandettini P.A., Wong E.C. (1997). A hypercapnia-based normalization method for improved spatial localization of human brain activation with fMRI. NMR Biomed..

[bb0025] Barch D.M., Burgess G.C., Harms M.P., Petersen S.E., Schlaggar B.L., Corbetta M., Glasser M.F., Curtiss S., Dixit S., Feldt C., Nolan D., Bryant E., Hartley T., Footer O., Bjork J.M., Poldrack R., Smith S., Johansen-Berg H., Snyder A.Z., Van Essen D.C. (2013). Function in the human connectome: task-fMRI and individual differences in behavior. NeuroImage.

[bb0030] Blockley N.P., Griffeth V.E., Simon A.B., Buxton R.B. (2013). A review of calibrated blood oxygenation level-dependent (BOLD) methods for the measurement of task-induced changes in brain oxygen metabolism. NMR Biomed..

[bb0035] Buxton R.B., Uludag K., Dubowitz D.J., Liu T.T. (2004). Modeling the hemodynamic response to brain activation. NeuroImage.

[bb0045] Chang C., Glover G.H. (2009). Relationship between respiration, end-tidal CO2, and BOLD signals in resting-state fMRI. NeuroImage.

[bb0040] Chang C., Cunningham J.P., Glover G.H. (2009). Influence of heart rate on the BOLD signal: the cardiac response function. NeuroImage.

[bb0050] Chen J.J., Pike G.B. (2010). MRI measurement of the BOLD-specific flow-volume relationship during hypercapnia and hypocapnia in humans. NeuroImage.

[bb0055] Chiarelli P.A., Bulte D.P., Wise R., Gallichan D., Jezzard P. (2007). A calibration method for quantitative BOLD fMRI based on hyperoxia. NeuroImage.

[bb0060] Chugani H.T., Phelps M.E., Mazziotta J.C. (1987). Positron emission tomography study of human brain functional development. Ann. Neurol..

[bb0065] Cohen E.R., Rostrup E., Sidaros K., Lund T.E., Paulson O.B., Ugurbil K., Kim S.G. (2004). Hypercapnic normalization of BOLD fMRI: comparison across field strengths and pulse sequences. NeuroImage.

[bb0075] Davis T.L., Kwong K.K., Weisskoff R.M., Rosen B.R. (1998). Calibrated functional MRI: mapping the dynamics of oxidative metabolism. Proc. Natl. Acad. Sci. U. S. A..

[bb0080] Deichmann R., Josephs O., Hutton C., Corfield D.R., Turner R. (2002). Compensation of susceptibility-induced BOLD sensitivity losses in echo-planar fMRI imaging. NeuroImage.

[bb0070] D'Esposito M., Zarahn E., Aguirre G.K., Rypma B. (1999). The effect of normal aging on the coupling of neural activity to the bold hemodynamic response. NeuroImage.

[bb0085] Di X., Kannurpatti S.S., Rypma B., Biswal B.B. (2013). Calibrating BOLD fMRI activations with neurovascular and anatomical constraints. Cereb. Cortex.

[bb0090] Donahue M.J., Dethrage L.M., Faraco C.C., Jordan L.C., Clemmons P., Singer R., Mocco J., Shyr Y., Desai A., O'Duffy A., Riebau D., Hermann L., Connors J., Kirshner H., Strother M.K. (2014). Routine clinical evaluation of cerebrovascular reserve capacity using carbogen in patients with intracranial stenosis. Stroke; a j. cereb. circulation.

[bb0095] Fera F., Yongbi M.N., van Gelderen P., Frank J.A., Mattay V.S., Duyn J.H. (2004). EPI-BOLD fMRI of human motor cortex at 1.5 T and 3.0 T: sensitivity dependence on echo time and acquisition bandwidth. J. Magn. Reson. Imaging JMRI.

[bb0100] Gaillard W.D., Grandin C.B., Xu B. (2001). Developmental aspects of pediatric fMRI: considerations for image acquisition, analysis, and interpretation. NeuroImage.

[bb0105] Garcia-Eulate R., Garcia-Garcia D., Dominguez P.D., Noguera J.J., De Luis E., Rodriguez-Oroz M.C., Zubieta J.L. (2011). Functional bold MRI: advantages of the 3 T vs. the 1.5 T. Clin. Imaging.

[bb0110] Gati J.S., Menon R.S., Ugurbil K., Rutt B.K. (1997). Experimental determination of the BOLD field strength dependence in vessels and tissue. Magn. Reson. Med. Off. j. Soc. Magn. Reson. Med. Soc. Magn. Reson. Med..

[bb0115] Glasser M.F., Sotiropoulos S.N., Wilson J.A., Coalson T.S., Fischl B., Andersson J.L., Xu J., Jbabdi S., Webster M., Polimeni J.R., Van Essen D.C., Jenkinson M. (2013). The minimal preprocessing pipelines for the Human Connectome Project. NeuroImage.

[bb0120] Grubb R.L., Raichle M.E., Eichling J.O., Ter-Pogossian M.M. (1974). The effects of changes in PaCO2 on cerebral blood volume, blood flow, and vascular mean transit time. Stroke; a j. cereb. circulation.

[bb0125] Handwerker D.A., Gazzaley A., Inglis B.A., D'Esposito M. (2007). Reducing vascular variability of fMRI data across aging populations using a breathholding task. Hum. Brain Mapp..

[bb0130] Hino J.K., Short B.L., Rais-Bahrami K., Seale W.R. (2000). Cerebral blood flow and metabolism during and after prolonged hypercapnia in newborn lambs. Crit. Care Med..

[bb0135] Hoge R.D. (2012). Calibrated FMRI. NeuroImage.

[bb0140] Hope T.M., Prejawa S., Parker J., Oberhuber M., Seghier M.L., Green D.W., Price C.J. (2014). Dissecting the functional anatomy of auditory word repetition. Front. Hum. Neurosci..

[bb0145] Horvath I., Sandor N.T., Ruttner Z., McLaughlin A.C. (1994). Role of nitric oxide in regulating cerebrocortical oxygen consumption and blood flow during hypercapnia. J. Cereb. Blood Flow Metab. off. j. Int. Soc. Cereb. Blood Flow Metab.

[bb0150] Huber L., Ivanov D., Krieger S.N., Streicher M.N., Mildner T., Poser B.A., Moller H.E., Turner R. (2014). Slab-selective, BOLD-corrected VASO at 7 Tesla provides measures of cerebral blood volume reactivity with high signal-to-noise ratio. Magn. Reson. Med. off. j. Soc. Magn. Reson. Med. Soc. Magn. Reson. Med..

[bb0155] Huettel S.A., McCarthy G. (2001). Regional differences in the refractory period of the hemodynamic response: an event-related fMRI study. NeuroImage.

[bb0160] Hutton C., Featherstone E., Weiskopf N. (2008). Cardio-respiratory effects on the phase in EPI.

[bb0165] Hutton C., Josephs O., Stadler J., Featherstone E., Reid A., Speck O., Bernarding J., Weiskopf N. (2011). The impact of physiological noise correction on fMRI at 7 T. NeuroImage.

[bb0170] Jones M., Berwick J., Hewson-Stoate N., Gias C., Mayhew J. (2005). The effect of hypercapnia on the neural and hemodynamic responses to somatosensory stimulation. NeuroImage.

[bb0175] Kalcher K., Boubela R.N., Huf W., Biswal B.B., Baldinger P., Sailer U., Filzmoser P., Kasper S., Lamm C., Lanzenberger R., Moser E., Windischberger C. (2013). RESCALE: voxel-specific task-fMRI scaling using resting state fluctuation amplitude. NeuroImage.

[bb0180] Kannurpatti S.S., Biswal B.B. (2008). Detection and scaling of task-induced fMRI-BOLD response using resting state fluctuations. NeuroImage.

[bb0185] Kannurpatti S.S., Biswal B.B., Hudetz A.G. (2002). Differential fMRI-BOLD signal response to apnea in humans and anesthetized rats. Magn. reson. med. off. j. Soc. Magn. Reson. Med. Soc. Magn. Reson. Med..

[bb0190] Kannurpatti S.S., Rypma B., Biswal B.B. (2012). Prediction of task-related BOLD fMRI with amplitude signatures of resting-state. Front. Syst. Neurosci..

[bb0195] Kastrup A., Kruger G., Glover G.H., Moseley M.E. (1999). Assessment of cerebral oxidative metabolism with breath holding and fMRI. Mag. reson. med. off. j. Soc. Magn. Reson. Med. Soc. Mag. Reson. Med..

[bb0200] Kaza E., Klose U., Lotze M. (2011). Comparison of a 32-channel with a 12-channel head coil: are there relevant improvements for functional imaging?. J. Magn. Reson. Imaging JMRI.

[bb0205] Kennerley A.J., Harris S., Bruyns-Haylett M., Boorman L., Zheng Y., Jones M., Berwick J. (2012). Early and late stimulus-evoked cortical hemodynamic responses provide insight into the neurogenic nature of neurovascular coupling. J. cereb. blood flow metab off. j. Int. Soc. Cereb. Blood Flow Metab.

[bb0210] Krasnow B., Tamm L., Greicius M.D., Yang T.T., Glover G.H., Reiss A.L., Menon V. (2003). Comparison of fMRI activation at 3 and 1.5 T during perceptual, cognitive, and affective processing. NeuroImage.

[bb0215] Kruger G., Kastrup A., Glover G.H. (2001). Neuroimaging at 1.5 T and 3.0 T: comparison of oxygenation-sensitive magnetic resonance imaging. Magn. Reson. Med. off. j. Soc. Magn. Reson. Med. Soc. Magn. Reson. Med..

[bb0220] Larson-Prior L.J., Oostenveld R., Della Penna S., Michalareas G., Prior F., Babajani-Feremi A., Schoffelen J.M., Marzetti L., de Pasquale F., Di Pompeo F., Stout J., Woolrich M., Luo Q., Bucholz R., Fries P., Pizzella V., Romani G.L., Corbetta M., Snyder A.Z. (2013). Adding dynamics to the Human Connectome Project with MEG. NeuroImage.

[bb0225] Lauritzen M. (2005). Reading vascular changes in brain imaging: is dendritic calcium the key?. Nat. Rev. Neurosci..

[bb0230] Li T.Q., Kastrup A., Takahashi A.M., Moseley M.E. (1999). Functional MRI of human brain during breath holding by BOLD and FAIR techniques. NeuroImage.

[bb0235] Lieberman M.D., Cunningham W.A. (2009). Type I and Type II error concerns in fMRI research: re-balancing the scale. Soc. Cogn. Affect. Neurosci..

[bb0240] Logothetis N.K. (2008). What we can do and what we cannot do with fMRI. Nature.

[bb0245] Logothetis N.K., Wandell B.A. (2004). Interpreting the BOLD signal. Annu. Rev. Physiol..

[bb0250] Mayhew J.E., Askew S., Zheng Y., Porrill J., Westby G.W., Redgrave P., Rector D.M., Harper R.M. (1996). Cerebral vasomotion: a 0.1-Hz oscillation in reflected light imaging of neural activity. NeuroImage.

[bb0255] McPherson R.W., Derrer S.A., Traystman R.J. (1991). Changes in cerebral CO2 responsivity over time during isoflurane anesthesia in the dog. J. Neurosurg. Anesthesiol..

[bb0260] Menon R.S., Ogawa S., Tank D.W., Ugurbil K. (1993). Tesla gradient recalled echo characteristics of photic stimulation-induced signal changes in the human primary visual cortex. Magn. Reson. Med. off. j. Soc. Magn. Reson. Med. Soc. Magn. Reson. Med.

[bb0265] Mohtasib R.S., Lumley G., Goodwin J.A., Emsley H.C., Sluming V., Parkes L.M. (2012). Calibrated fMRI during a cognitive Stroop task reveals reduced metabolic response with increasing age. NeuroImage.

[bb0270] Mueller S., Wang D., Fox M.D., Yeo B.T., Sepulcre J., Sabuncu M.R., Shafee R., Lu J., Liu H. (2013). Individual variability in functional connectivity architecture of the human brain. Neuron.

[bb0275] Murphy K., Harris A.D., Wise R.G. (2011). Robustly measuring vascular reactivity differences with breath-hold: normalising stimulus-evoked and resting state BOLD fMRI data. NeuroImage.

[bb0280] Nilsson H., Aalkjaer C. (2003). Vasomotion: mechanisms and physiological importance. Mol. Interv..

[bb0285] Parker Jones O., Prejawa S., Hope T.M., Oberhuber M., Seghier M.L., Leff A.P., Green D.W., Price C.J. (2014). Sensory-to-motor integration during auditory repetition: a combined fMRI and lesion study. Front. Hum. Neurosci..

[bb0290] Porret C.A., Stergiopulos N., Hayoz D., Brunner H.R., Meister J.J. (1995). Simultaneous ipsilateral and contralateral measurements of vasomotion in conduit arteries of human upper limbs. Am. J. Physiol..

[bb0295] Price C.J., Seghier M.L., Leff A.P. (2010). Predicting language outcome and recovery after stroke: the PLORAS system. Nat. Rev. Neurol..

[bb0300] Prisman E., Slessarev M., Han J., Poublanc J., Mardimae A., Crawley A., Fisher J., Mikulis D. (2008). Comparison of the effects of independently-controlled end-tidal PCO(2) and PO(2) on blood oxygen level-dependent (BOLD) MRI. J. Magn. Reson. Imaging JMRI.

[bb0305] Richter W., Richter M. (2003). The shape of the fMRI BOLD response in children and adults changes systematically with age. NeuroImage.

[bb0310] Schechner J.S., Braverman I.M. (1992). Synchronous vasomotion in the human cutaneous microvasculature provides evidence for central modulation. Microvasc. Res..

[bb0315] Shmueli K., van Gelderen P., de Zwart J.A., Horovitz S.G., Fukunaga M., Jansma J.M., Duyn J.H. (2007). Low-frequency fluctuations in the cardiac rate as a source of variance in the resting-state fMRI BOLD signal. NeuroImage.

[bb0320] Siesjo B.K. (1980). Cerebral metabolic rate in hypercarbia—a controversy. Anesthesiology.

[bb0325] Sirotin Y.B., Das A. (2009). Anticipatory haemodynamic signals in sensory cortex not predicted by local neuronal activity. Nature.

[bb0330] Slaaf D.W., Vrielink H.H., Tangelder G.J., Reneman R.S. (1988). Effective diameter as a determinant of local vascular resistance in presence of vasomotion. Am. J. Physiol..

[bb0335] Thomason M.E., Burrows B.E., Gabrieli J.D., Glover G.H. (2005). Breath holding reveals differences in fMRI BOLD signal in children and adults. NeuroImage.

[bb0340] Triantafyllou C., Hoge R.D., Krueger G., Wiggins C.J., Potthast A., Wiggins G.C., Wald L.L. (2005). Comparison of physiological noise at 1.5 T, 3 T and 7 T and optimization of fMRI acquisition parameters. NeuroImage.

[bb0345] Tsvetanov K.A., Henson R.N., Tyler L.K., Davis S.W., Shafto M.A., Taylor J.R., Williams N., Cam C., Rowe J.B. (2015). The effect of ageing on fMRI: correction for the confounding effects of vascular reactivity evaluated by joint fMRI and MEG in 335 adults. Hum. Brain Mapp..

[bb0350] Van den Aardweg J.G., Karemaker J.M. (2002). Influence of chemoreflexes on respiratory variability in healthy subjects. Am. J. Respir. Crit. Care Med..

[bb0355] Villringer A., Dirnagl U. (1995). Coupling of brain activity and cerebral blood flow: basis of functional neuroimaging. Cerebrovasc. Brain Metab. Rev..

[bb0360] Weiskopf N., Hutton C., Josephs O., Deichmann R. (2006). Optimal EPI parameters for reduction of susceptibility-induced BOLD sensitivity losses: a whole-brain analysis at 3 T and 1.5 T. NeuroImage.

[bb0365] Wiggins G.C., Triantafyllou C., Potthast A., Reykowski A., Nittka M., Wald L.L. (2006). 32-channel 3 Tesla receive-only phased-array head coil with soccer-ball element geometry. Magn. Reson. Med. off. j. Soc. Magn. Reson. Med. Soc. Magn. Reson. Med..

[bb0375] Wise R.G., Ide K., Poulin M.J., Tracey I. (2004). Resting fluctuations in arterial carbon dioxide induce significant low frequency variations in BOLD signal. NeuroImage.

[bb0370] Wise R.G., Harris A.D., Stone A.J., Murphy K. (2013). Measurement of OEF and absolute CMRO2: MRI-based methods using interleaved and combined hypercapnia and hyperoxia. NeuroImage.

[bb0380] Xu F., Uh J., Brier M.R., Hart J., Yezhuvath U.S., Gu H., Yang Y., Lu H. (2011). The influence of carbon dioxide on brain activity and metabolism in conscious humans. J. Cereb. Blood Flow Metab. off. j. Int. Soc. Cereb. Blood Flow Metab.

[bb0385] Yablonskiy D.A. (2011). Cerebral metabolic rate in hypercapnia: controversy continues. J. Cereb. Blood Flow Metab. off. j. Int. Soc. Cereb. Blood Flow Metab.

[bb0390] Yang S.P., Krasney J.A. (1995). Cerebral blood flow and metabolic responses to sustained hypercapnia in awake sheep. J. Cereb. Blood Flow Metab. off. j. Int. Soc. Cereb. Blood Flow Metab.

[bb0395] Yang Y., Wen H., Mattay V.S., Balaban R.S., Frank J.A., Duyn J.H. (1999). Comparison of 3D BOLD functional MRI with spiral acquisition at 1.5 and 4.0 T. NeuroImage.

[bb0400] Yezhuvath U.S., Lewis-Amezcua K., Varghese R., Xiao G., Lu H. (2009). On the assessment of cerebrovascular reactivity using hypercapnia BOLD MRI. NMR Biomed..

[bb0405] Zang Y.F., He Y., Zhu C.Z., Cao Q.J., Sui M.Q., Liang M., Tian L.X., Jiang T.Z., Wang Y.F. (2007). Altered baseline brain activity in children with ADHD revealed by resting-state functional MRI. Brain Dev..

[bb0410] Zappe A.C., Uludag K., Oeltermann A., Ugurbil K., Logothetis N.K. (2008). The influence of moderate hypercapnia on neural activity in the anesthetized nonhuman primate. Cereb. Cortex.

